# The Alternative Pathway Is Necessary and Sufficient for Complement Activation by Anti-THSD7A Autoantibodies, Which Are Predominantly IgG4 in Membranous Nephropathy

**DOI:** 10.3389/fimmu.2022.952235

**Published:** 2022-07-07

**Authors:** Pallavi Manral, Tiffany N. Caza, Aaron J. Storey, Laurence H. Beck, Dorin-Bogdan Borza

**Affiliations:** ^1^ Department of Microbiology, Immunology and Physiology, Meharry Medical College, Nashville, TN, United States; ^2^ Arkana Laboratories, Little Rock, AR, United States; ^3^ Department of Biochemistry and Molecular Biology, University of Arkansas for Medical Sciences, Little Rock, AR, United States; ^4^ Department of Medicine (Nephrology), Boston University School of Medicine and Boston Medical Center, Boston, MA, United States

**Keywords:** complement activation, alternative pathway of complement, membranous nephropathy, THSD7A (thrombospondin type 1 domain-containing protein 7A), IgG4 antibodies

## Abstract

Membranous nephropathy (MN) is an immune kidney disease characterized by glomerular subepithelial immune complexes (ICs) containing antigen, IgG, and products of complement activation. Whereas proteinuria is caused by complement-mediated podocyte injury, the pathways of complement activation remain controversial due to the predominance of IgG4 in ICs, an IgG subclass considered unable to activate complement. THSD7A, a transmembrane protein expressed on podocytes, is the target autoantigen in ~3% of cases of primary MN. In this study, we analyzed sera from 16 patients with THSD7A-associated MN with regard to the anti-THSD7A IgG subclasses and their ability to fix complement *in vitro*. The serum concentration of anti-THSD7A IgG varied over two orders of magnitude (1.3-243 μg/mL). As a relative proportion of all IgG anti-THSD7A, IgG4 was by far the most abundant subclass (median 79%), followed by IgG1 (median 11%). IgG4 was the dominant subclass of anti-THSD7A antibodies in 14 sera, while IgG1 was dominant in one and co-dominant in another. One quarter of MN sera additionally contained low levels of anti-THSD7A IgA1. ICs formed by predominantly IgG4 anti-THSD7A autoantibodies with immobilized THSD7A were relatively weak activators of complement *in vitro*, compared to human IgG1 and IgG3 mAbs used as positive control. Complement deposition on THSD7A ICs was dose-dependent and occurred to a significant extent only at relatively high concentration of anti-THSD7A IgG. C3b fixation by THSD7A ICs was completely abolished in factor B-depleted sera, partially inhibited in C4-depleted sera, unchanged in C1q-depleted sera, and also occurred in Mg-EGTA buffer. These results imply that THSD7A ICs predominantly containing IgG4 activate complement at high IgG4 density, which strictly requires a functional alternative pathway, whereas the classical and lectin pathways are dispensable. These findings advance our understanding of how IgG4 antibodies activate complement.

## Introduction

Membranous nephropathy (MN), one of the leading causes of nephrotic syndrome in adults, is an antibody-mediated kidney disease clinically characterized by proteinuria, often heavy and persistent. MN is a disease of heterogeneous etiology, defined histologically by immune complexes (ICs) deposited on the subepithelial side of glomerular basement membrane (GBM), together with GBM thickening and podocyte foot process effacement, but without proliferative changes. The most prevalent form is the so-called “primary” MN, an autoimmune disease in which IgG autoantibodies (autoAbs) form subepithelial ICs with autoantigens expressed on podocyte cell surface (1, 2). About 70% of patients with primary MN have autoAbs targeting phospholipase A2 receptor (PLA2R), and an additional 3-5% have autoAbs targeting thrombospondin type-1 domain-containing 7A (THSD7A) (3, 4). Serologic assays established for both PLA2R and THSD7A show that circulating autoAb levels correlate to disease activity, are useful for therapeutic monitoring, and inform prognosis (5–8). Recent studies have identified additional autoantigens or biomarkers implicated in MN, including neural epidermal growth factor-like 1 (9), exostosin 1/2 (10), serine protease HTRA1 (11), neural cell adhesion molecule-1 (12), semaphorin 3B (13), protocadherin 7 (14), transforming growth factor beta receptor 3 (15), and contactin (16).

In the current paradigm for the pathogenesis of MN based on studies in passive Heymann nephritis, complement activation by subepithelial ICs leading to sublethal podocyte injury by C5b-9 is a major effector mechanism of proteinuria (17). In human MN, complement activation products are abundant in subepithelial deposits. Nonetheless, because the autoAbs implicated in PLA2R- and THSD7A-associated MN are predominantly of the IgG4 subclass (7, 17–20), how ICs activate complement in MN remains a conundrum (21). IgG4 does not bind C1q and is considered unable to activate complement, at least not *via* the classical pathway (22–24). Yet, ICs formed by model monoclonal human IgG4 antibodies are capable of activating complement *via* the alternative pathway under certain circumstances, i.e. at high IgG concentrations and high epitope density (25–27).

In PLA2R-associated MN, it has been reported that altered glycosylation (degalactosylation) of IgG4 autoAbs promotes the binding of mannose-binding lectin (MBL) and complement activation *via* the lectin pathway, which mediates injury of human podocytes in culture (28). One caveat is that MBL binding to IgG4 purified by acid elution may be artifactual, given that acid denaturation of human IgG was reported to increase MBL binding to an even greater extent than IgG degalactosylation (29). PLA2R-associated MN can in fact occur in individuals with complete MBL deficiency, in whom complement is activated solely *via* the alternative pathway, implying that the lectin pathway is dispensable (30). Another study has found that the classical pathway initiates and the alternative pathway amplifies complement activation by model immune complexes formed by anti-PLA2R1 autoAbs *in vitro*, suggesting a role for non-IgG4 autoAbs (31). Finally, a proteomic analysis of complement proteins in glomeruli from PLA2R1-associated MN suggests that both classical/lectin and alternative pathways drive and contribute to the activation of terminal pathway (32).

In THSD7A-associated MN, there is agreement that autoAbs are predominantly IgG4, but the relative proportion of other IgG subclasses is less clear. Some studies have shown low abundance of IgG1, IgG2 and IgG3 anti-THSD7A (4, 20), yet others have shown relatively intense staining for IgG1, IgG2 and IgG3, in addition to IgG4 (33). These differences are likely due to the use of qualitative assays, without standardization of subclass-specific secondary antibodies. Since the four IgG subclasses show wide variation in regard to their effector properties (23), the goal of this study was to quantitatively measure the serum levels of anti-THSD7A IgG subclasses and investigate the functional properties of anti-THSD7A autoAbs, i.e, their ability to activate complement *in vitro*.

## Materials and methods

### Materials

The recombinant human THSD7A was purchased from R&D (9524-TH, Minneapolis, Minnesota). Fresh-frozen normal human serum and complement C1q-, C4-, or factor B-depleted human sera in which more than 99% of the functional protein was depleted were from Complement Technologies (Tyler, Texas). Recombinant monoclonal human IgG1, IgG2, IgG3 and IgG4 anti-TNFα antibodies were purchased from InvivoGen (San Diego, California). Recombinant monoclonal human IgG1, IgG2, IgG3, IgG4, and IgA1 anti-GFP antibodies were from BioRad (Hercules, California). Recombinant human TNFα and *A. victoria* GFP were purchased from Millipore (Burlington, Massachusetts) and Abcam, respectively.

Serum samples from THSD7A-associated MN patients had been collected Boston Medical Center, or obtained from banked residual sera from patients who underwent indirect immunofluorescence testing for anti-THSD7A antibodies at Arkana Laboratories. All serum samples were collected with informed consent under IRB-approved protocols at each institution. All included patients had a positive test of 1:10 or greater by the Euroimmun indirect immunofluorescence test (IIFT). The demographic and clinical characteristics are summarized in **Table 1**. The study cohort comprised 6 males and 10 females and included 7 Caucasians, 5 African-Americans and 2 Asians. The average age ± SD was 60 ± 20 years. The median values for proteinuria, serum albumin, and serum creatinine were 7.5 g/day, 2.7 g/dL, and 0.87 mg/dL, respectively. The results of routine IF staining in the biopsy were available for 12 cases, shown in **Table 2**. All biopsies showed positive staining for IgG and C3. IgA, IgM and C1q staining were negative, except for one case with weak IgM and another with weak IgA staining.

**Table 1:** Demographic and clinical characteristics of anti-THSD7A positive patients.

Patient characteristics	Result
Number of cases	16
Sex: M/F	6 (37.5%):10 (62.5%)
Age (yr)	61 (47 – 77)
Ethnicity: White:	7 (43.8%)
African-American:	5 (31.2%)
Asian:	2 (12.5%)
Unknown	2 (12.5%)
Proteinuria (g/day)	7.5 (2.8 – 10.4)
Serum albumin (g/dL)	2.7 (1.89 – 3.6)
Serum creatinine (mg/dL)	0.87 (0.80 – 1.47)

Values are median (interquartile range) or number (%).

**Table 2:** The results of immunofluorescence staining in the MN renal biopsies.

Patient #	IgG	IgA	IgM	C3	C1q
MN-1	3+	0	0	1+	0
MN-2	4+	1+	0	3+	0
MN-3	4+	0	0	2+	0
MN-4	3+	0	0	1+	0
MN-5	3+	0	trace	1+	0
MN-6	2+	0	0	1+	0
MN-7	4+	0	0	2+	0
MN-8	1+	N.D.^a^	N.D.^a^	N.D.^a^	N.D.^a^
MN-9	positive	0	0	positive	0
MN-11	3+	0	1+	1+	0
MN-13	3+	0	0	1+	0
MN-14	3+	0	0	2+	N.D.
MN-15	4+	0	0	3+	0

a N.D., Not determined; no glomeruli in IF sample.

### Quantitative Analysis of Serum Levels of Anti-THSD7A AutoAb Subclasses by ELISA

Serum antibodies binding to THSD7A were assayed by ELISA. Briefly, 96-well Nunc MaxiSorp microtiter plates (ThermoFisher Scientific, Waltham, Massachusetts) were coated overnight with THSD7A (150 ng per well) in carbonate-bicarbonate buffer, pH 9.6. After blocking with 1% bovine serum albumin (BSA), the wells were incubated for 1 hour with patient or control sera diluted 1/100. Some samples required further dilution (1/200-1/1,600) for IgG4 detection. Secondary antibodies were horseradish peroxidase-conjugated sheep anti-human IgG1-4 (The Binding Site, Birmingham, UK), goat anti-human IgA, and mouse anti-human IgA1 and IgA2 (Southern Biotech, Birmingham, Alabama). Plates were developed with TMB Microwell peroxidase substrate (VWR, Radnor, Pennsylvania), and absorbance was read at 650 nm with an ELISA plate reader (BioRad). A blank correction was applied to all values by subtracting the OD in antigen-coated wells incubated with buffer instead of serum. In parallel experiments, standard curves for human IgG1-IgG4 were generated by incubating known concentrations of recombinant human monoclonal IgG1-IgG4 anti-TNFα (2-500 ng/ml) in wells coated with recombinant TNFα (150 ng/well in carbonate buffer pH 9.6). A standard curve for IgA was generated by incubating human IgA1 anti-GFP mAb in wells coated with GFP. The serum concentration of anti-THSD7A IgG subclasses or IgA was interpolated from the standard curves by a non-linear regression assuming a one-site specific binding model.

### *In Vitro* Complement Activation Assay

We developed a new functional assay to investigate the ability of anti-THSD7A autoAbs to fix complement. ELISA plates were coated with THSD7A protein (200 ng in 50 μl PBS), overnight at 4°C. The plates were next blocked with 1% BSA in phosphate-buffered saline (PBS) for 1 hour at room temperature, washed, and then incubated for 1 hour with sera from MN patients or non-MN controls (diluted 1/25 in PBS with 0.1% BSA and 0.05% Tween-20/PBS). Next, plates were washed again and incubated for 30 minutes at 37°C with fresh-frozen normal human serum (NHS) diluted 10% in gelatin-veronal buffer (GVB) containing 0.15 mM Ca^2+^ and 0.5 mM Mg^2+^, in which all three complement activation pathways are functional. In some experiments, NHS was diluted in GVB with 5 mM MgEGTA (which inhibits the classical and lectin pathways), or in GVB with 10 mM EDTA (which inhibits all complement activation pathways). In other experiments, C1q-, C4-, and factor B-depleted sera were used instead of NHS. After a wash step, deposition of complement activation products was assayed. The deposition of C3b and its proteolytic inactivation product, iC3b, was detected with sheep anti-human C3c-HRP (BioRad, 2222-6604P) diluted 1:1000. C5b-9 (terminal complement complex, TCC) deposition was detected with mouse anti-human TCC mAb (HM 2167S, Hycult Biotech) diluted 1:500. Properdin (FP) was detected using mouse anti-human properdin mAb (Hycult Biotech, HM2283S) diluted 1:500. C1q and factor B (FB) were detected using goat anti-human C1q and goat anti-human FB antibodies (Quidel), diluted 1:2000. C4 was detected using goat anti-human C4 antibodies (Complement Technologies, A205) diluted 1:40000. The secondary antibodies were goat anti-mouse IgG-HRP diluted 1:2000 and donkey anti-goat IgG-HRP diluted 1:10000 (both from Invitrogen). The absorbance at 650 nm was read after color development with peroxidase substrate. In parallel assays, complement activation was also performed in wells coated with GFP (200 ng/50 μl in carbonate buffer, pH 9.6) and incubated with various concentrations (1-5 μg/mL) of human monoclonal anti-GFP IgG1к, IgG3к and IgG4ʎ.

### Statistical Analyses

Analyses were performed using GraphPad Prism 8.4 software (San Diego, CA). The significance of differences among groups was determined by one-way ANOVA followed by Bonferroni’s test for pairwise comparisons, or by repeated measures ANOVA with the Geisser-Greenhouse correction, followed by Dunnet’s multiple comparisons test. Other statistical analyses are described in the text or the figure legends. A multiplicity-adjusted value of *P* < 0.05 was considered statistically significant.

## Results

### IgG4 Is the Dominant Subclass of Anti-THSD7A AutoAbs

**Figure 1:**
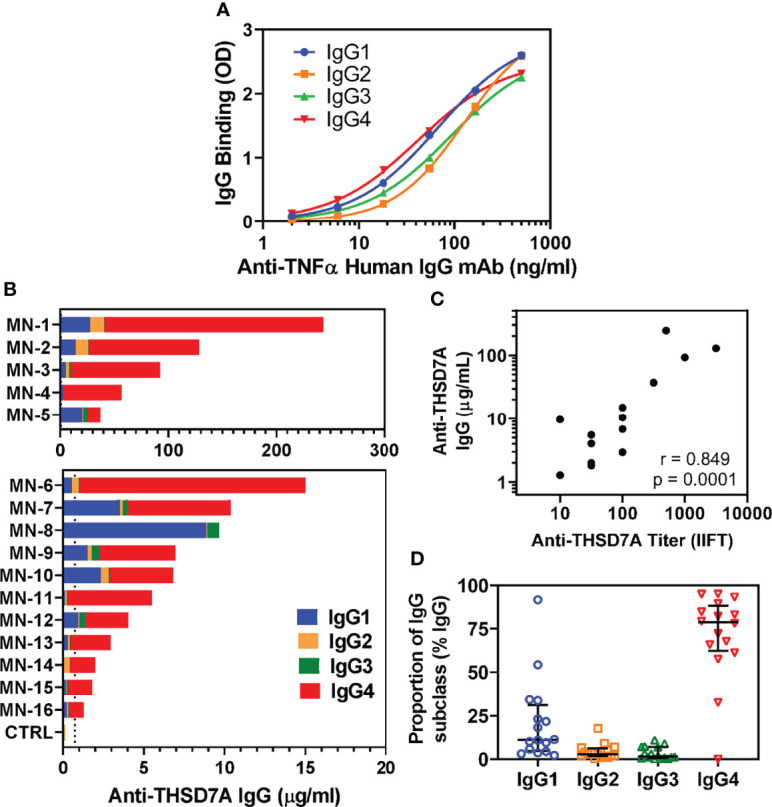
Analysis of IgG subclasses of anti-THSD7A autoAbs. **(A)** Standard curves show the concentration dependence for the binding of recombinant human anti-TNFα IgG1 (blue), IgG2 (orange), IgG3 (green) and IgG4 (red) to wells coated with TNFα antigen. **(B)** Sera from 16 patients with THSD7A-associated MN were analyzed by ELISA for IgG autoAb subclasses binding to immobilized THSD7A. The serum concentrations of anti-THSD7A IgG1 (blue), IgG2 (orange), IgG3 (green) and IgG4 (red) in each MN serum were interpolated from standard curves using recombinant human IgG1-IgG4 mAbs anti-TNFα. Sera were designated MN-1 to MN-16 in decreasing order of their levels of anti-THSD7A IgG, calculated as the sum of the concentrations of all four IgG subclasses. **(C)** The correlation between serum levels of anti-THSD7A IgG measured by ELISA and the anti-THSD7A titers measured by indirect immunofluorescence test (IIFT) was significant (Pearson r=0.849, p=0.0001). **(D)** The relative proportion of IgG subclasses in MN sera was calculated by dividing the serum concentration of each subclass to sum of concentrations for all four IgG subclasses of autoAbs. The error bars show the median and the interquartile range.

Circulating autoAbs were analyzed in 16 sera from patients with THSD7A-associated MN along with four normal sera used as controls. To quantitatively measure the levels of anti-THSD7A subclasses, standard/calibration curves were generated using serial dilutions of anti-TNFα human IgG1-IgG4 mAbs bound to immobilized TNFα (**Figure 1A**). The total concentration of anti-THSD7A IgG varied over a broad range (1.3-243 μg/mL), spanning over two orders of magnitude (**Figure 1B**), consistent with previous reports (8). The anti-THSD7A IgG levels measured by ELISA and the anti-THSD7A titers measured by a commercial indirect immunofluorescence test (IIFT) were significantly correlated (**Figure 1C**). The mean ± SD for anti-THSD7A IgG in control sera was 0.14 ± 0.18 μg/ml. Among IgG autoAbs, the relative proportion of IgG4 was the highest (median 79%, interquartile range IQR 62%-88%), followed by IgG1 (median 11%, IQR 4.8%-31%), while the proportions of IgG2 (median 2.8%, IQR 1.8%-6.2%), and IgG3 (median 1.5%, IQR 0.7%-7%) were low (**Figure 1D**). Although IgG4 was the dominant subclass of anti-THSD7A in the majority of MN sera (14/16), IgG1 was dominant in one case (MN-8, diagnosed as secondary MN based on the EM findings in the biopsy), and co-dominant in another (MN-5). For four cases for which IgG subclass staining was performed on the kidney biopsy, the intensity of the staining showed a good correlation with the results of serologic assays for anti-THSD7A IgG subclasses (**Table 3**).

**Table 3:** The correlation between IgG subclasses in serum and in the renal biopsy.

Patient #	IgG1	IgG2	IgG3	IgG4
MN-3	2.5+ (5.3)	2+ (2.6)	1+ (1.7)	4+ (82.7)
MN-4	3+ (2.6)	0 (0.11)	0 (0.13)	3+ (53.9)
MN-6	1+ (0.55)	1+ (0.41)	0 (0.04)	2+ (14.0)
MN-8	1+ (8.9)	0 (0.08)	trace (0.71)	0 (0.0)
MN-9	ND (1.5)	ND (0.25)	ND (0.48)	3+ (4.7)

The values represent the intensity of IF staining (serum anti-THSD7A IgG subclass level in μg/ml).

**Figure 2:**
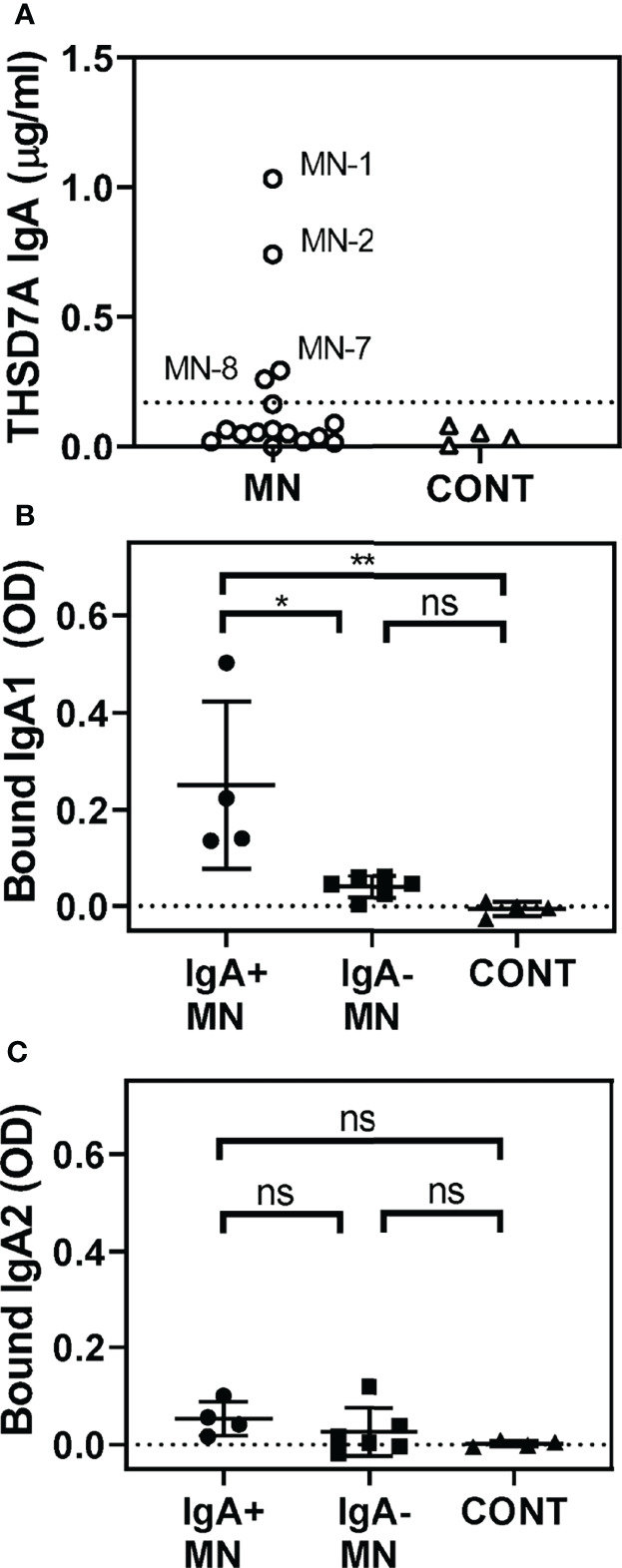
Identification of IgA anti-THSD7A autoAbs. **(A)** Quantitative analysis of anti-THSD7A IgA in MN sera (circles). The threshold for positivity, depicted by the dotted line, was defined as the mean + 4SD of control sera (tringles). B-C. IgA-positive MN sera (circles) show significantly higher binding to THSD7A of IgA1 **(B)** but not IgA2 **(C)**, compared to IgA-negative MN sera (squares) or control sera (triangles). The significance of differences among groups was analyzed by one-way ANOVA follow by Bonferroni’s test for multiple comparison (ns, not significant; *p<0.05; **p<0.01).

We further analyzed the possible presence of other isotypes of anti-THSD7A autoAbs. Interestingly, a quarter of MN sera (4/16) were positive for IgA anti-THSD7A (**Figure 2A**). However, the levels of IgA autoAbs were relatively low (0.26-1.03 μg/mL), corresponding to 0.2%-0.5% of the levels of IgG autoAbs in the respective sera. Further analysis of subclasses of anti-THSD7A IgA revealed the presence of IgA1 (**Figure 2B**) but not IgA2 (**Figure 2C**). IgM or IgE anti-THSD7A were not detected (data not shown).

### Anti-THSD7A Antibodies Bound to THSD7A Antigen Activate Complement *In Vitro*

**Figure 3:**
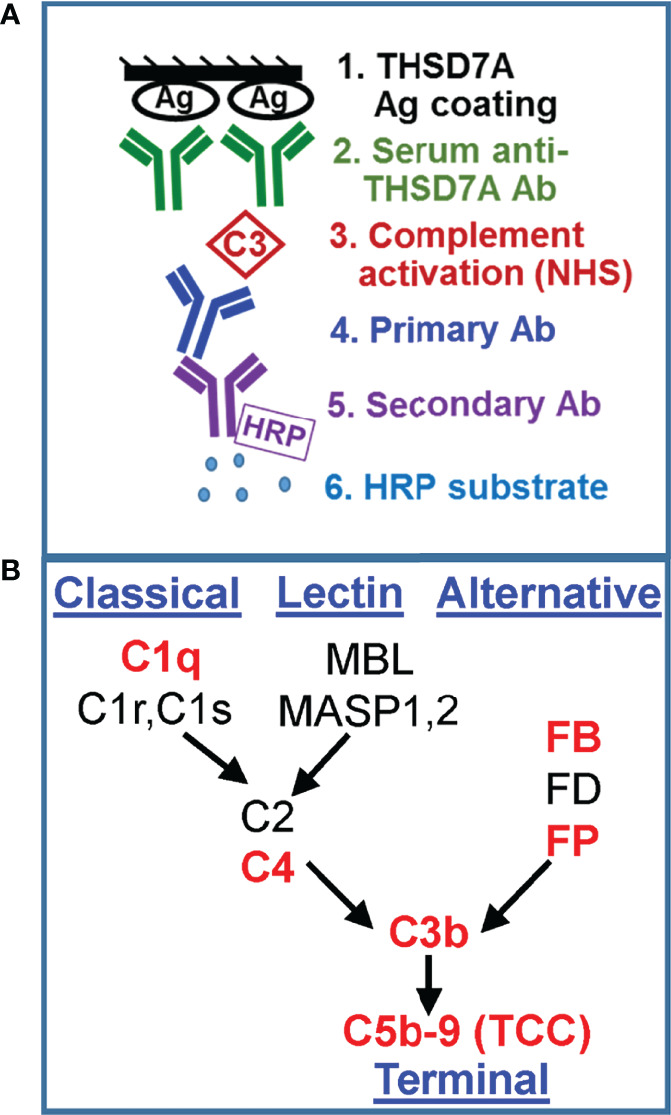
Development of a functional assay for complement fixation by THSD7A ICs. **(A)** Schematic representation of the *in vitro* assay for complement activation. **(B)** Simplified diagram of the complement activation pathways. The complement proteins for which the binding to ICs was assayed in this study are shown in red.

**Figure 4:**
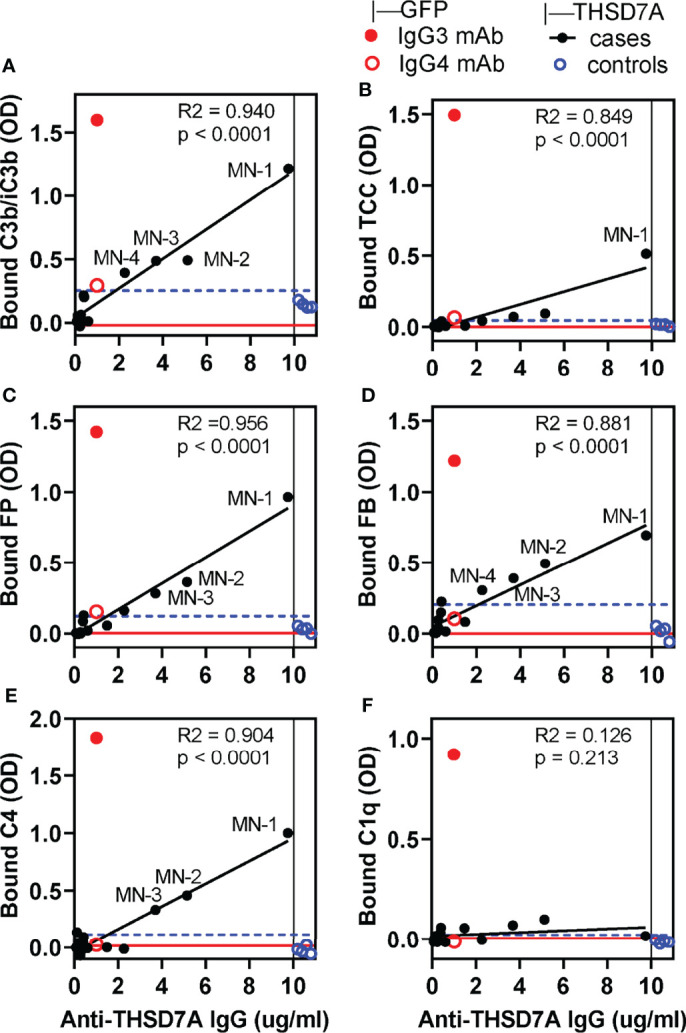
*In vitro* complement activation by THSD7A ICs under conditions in which all activation pathways are active. Complement activation was performed using 10% normal human serum (NHS) diluted in GVB++ buffer containing Ca^2+^ and Mg^2+^ in wells coated with THSD7A (200 ng/well) and then incubated with sera MN-1 through MN-14 (solid black circles) or control sera (open blue circles), all diluted 1/25. For each sample, the x-coordinate indicates the concentration of anti-THSD7A IgG in the diluted MN serum. In parallel experiments, activation was performed on reference ICs prepared by incubating human IgG3 (1 μg/ml, solid red circle) and IgG4 (1 μg/ml, open red circle) anti-GFP mAbs in GFP-coated wells (200 ng/well). Graphs depict the binding of antibodies specific to C3b/iC3b **(A)**, C5b-9/TCC **(B)**, FP **(C)**, FB **(D)**, C4 **(E)**, and C1q **(F)**. A blank correction was performed by subtracting the OD value on immobilized antigen incubated with PBS from the OD value of immobilized antigen incubated with antibody. The threshold for positivity (blue dashed line) was calculated as the mean OD of the control sera + 4 SD. Labels indicate MN sera which showed complement deposition above the positivity threshold. The OD values for MN sera were fitted to the concentration of anti-THSD7A IgG in each sample (values taken from Figure 1 and divided by the dilution factor) by simple linear regression (black line). The red line indicates the baseline obtained by performing the complement activation assays using 10% heat-inactivated NHS in GVB++ as a source of complement.

We developed a new functional assay to investigate the ability of serum anti-THSD7A autoAbs to activate complement *in vitro* (**Figure 3A**). First, immobilized THSD7A was incubated with diluted MN sera to form plate-bound ICs. In control experiments performed in parallel, human anti-GFP IgG3 and IgG4 mAbs were bound to immobilized GFP to prepare reference ICs. Next, 10% normal human serum diluted in buffer containing Ca^2+^ and Mg^2+^ was added as a source of complement, and then complement activation products deposited onto the ICs were detected using specific antibodies (**Figure 3B**). We assayed the binding of C3b/iC3b as a marker of overall complement activation *via* all pathways (**Figure 4A**), C5b-9 as a marker of terminal complement pathway activation (**Figure 4B**), properdin (FP; **Figure 4C**) and factor B (FB, **Figure 4D**) as markers of the alternative pathway activation, C4 as a marker of classical and lectin pathways activation (**Figure 4E**), and C1q as a marker of classical pathway activation (**Figure 4F**).

In general, the amount of C3b/iC3b, properdin, factor B and C4 bound to THSD7A ICs varied linearly with the concentration of anti-THSD7A IgG in each serum sample. Nonetheless, significant binding (i.e., above the cutoff value set at 4 SD above the mean of control sera) was only observed for MN sera in which the anti-THSD7A IgG concentration —adjusted for the dilution factor—was greater than 3.7 μg/mL (i.e. MN-1, MN-2, and MN-3). Complement deposition on THSD7A ICs formed by serum MN-4 (which contained 2.3 μg/mL anti-THSD7A IgG when diluted 1/25) was borderline. For all other MN sera with lower titers of anti-THSD7A IgG, complement fixation was negligible, comparable to control sera. Significant activation of the terminal pathway, as judged from C5b9 deposition, was only observed for serum MN-1, which had the highest titer of anti-THSD7A IgG (**Figure 4B**). This suggests that the activation of terminal complement pathway requires higher density of IgG4 in ICs that the activation at the level of C3. Finally, the binding of C1q to THSD7A ICs was negligible, which is consistent with the negative C1q staining in the MN patient biopsies. Overall, the deposition of factor B and properdin suggests the activation of the alternative pathway, while deposition of C4 in the absence of C1q suggests (but not prove) lectin pathway.

### The Alternative Pathway Is Essential for Complement Activation by THSD7A ICs

**Figure 5:**
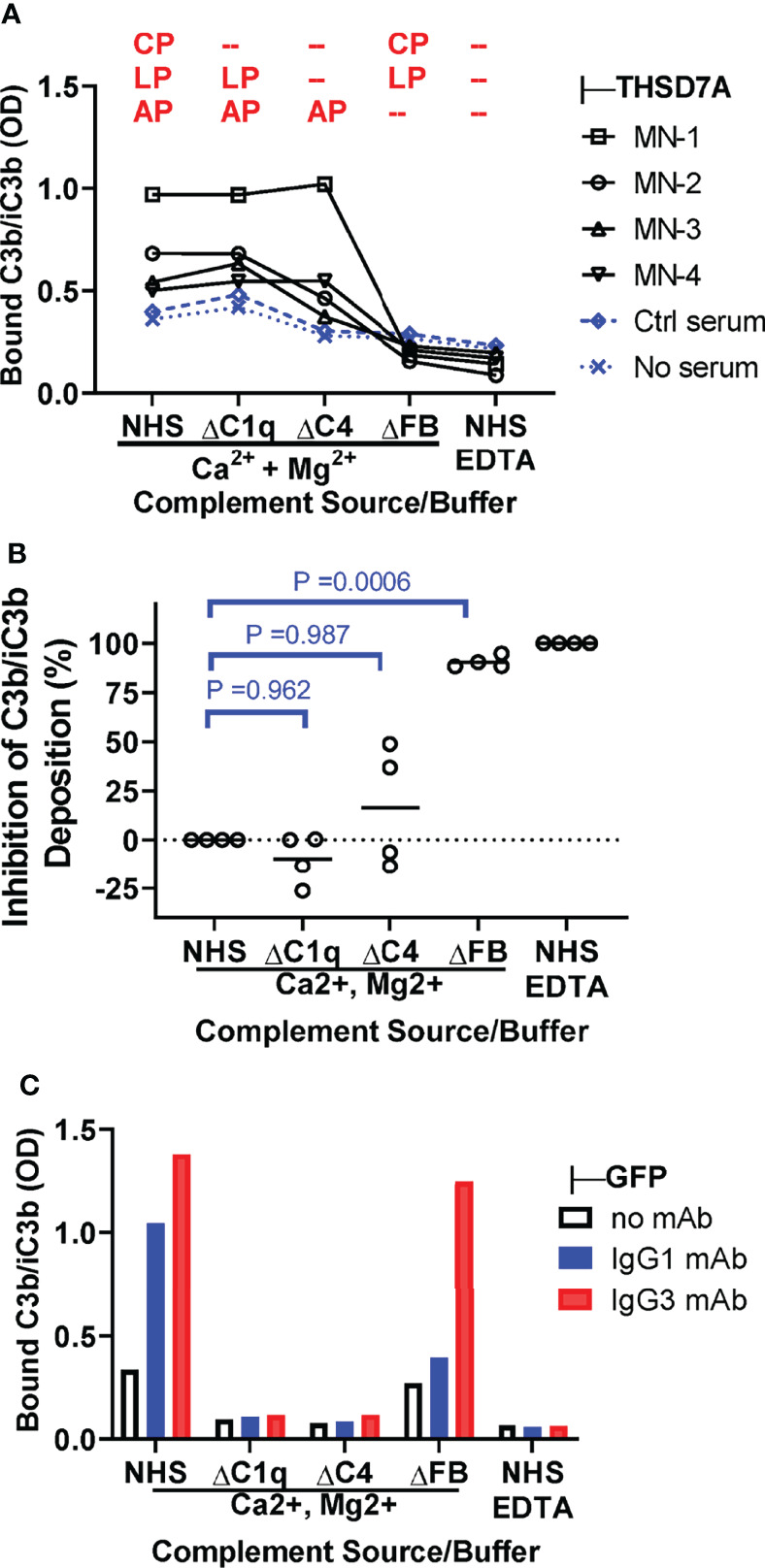
The alternative pathway is essential for complement activation by THSD7A ICs. **(A)** Plot depicting the deposition of C3b/iC3b (expressed as OD) onto THSD7A ICs formed by MN1-MN4 sera (black symbols) or in THSD7A-coated wells incubated with buffer or control serum (blue symbols). Complement activation was performed in 10% normal human serum (NHS), C1q-depleted serum (ΔC1q), C4-depleted serum (ΔC4), and FB-depleted serum (ΔFB) in buffer containing Ca2+ and Mg2+, as well as 10% NHS in EDTA buffer. The complement activation pathway active under each condition are shown in red (CP, classical pathway; LP, lectin pathway, AP, alternative pathway). **(B)** The relative inhibition of C3b/iC3b deposition of onto THSD7A ICs formed by MN1-MN4 sera was calculated for C1q-, C4- and FB-depleted sera on a scale from 0% inhibition (10% NHS in buffer containing Ca2+ and Mg2+) to 100% inhibition (10% NHS in EDTA buffer). C3b/iC3b deposition was significantly inhibited in FB-depleted serum lacking a functional alternative pathway (p=0.0006, one-way repeated measures ANOVA followed by *post-hoc* Dunnet test). **(C)** Deposition of C3b/iC3b on human monoclonal IgG1 and IgG3 anti-GFP (1 μg/mL each) bound to TNFα was measured after incubation with 10% NHS or C1q-, C4 and FB-depleted sera in buffer containing Ca2+ and Mg2+ or in 10% NHS in EDTA-containing buffer as source of complement.

We next investigated the contribution of specific pathways to complement activation by THSD7A ICs by measuring the deposition of C3b/iC3b from C1q-depleted serum (lacking a functional classical pathway), C4-depleted serum (lacking functional classical and lectin pathways) and FB-depleted serum (lacking a functional alternative pathway), as compared to normal human serum as source of complement (**Figure 5A**). C3b/iC3b deposition was almost completely abolished in FB-deficient serum (88-95% inhibition), largely unaffected in C1q-depleted serum, and partially reduced (35-50% inhibition) in C4-depleted serum for some but not all MN sera (**Figure 5B**). This result indicates that the alternative pathway is essential for complement activation by IgG4-dominant THSD7A ICs, while the classical pathway is dispensable, and the lectin pathway may play a limited role. By contrast, in control experiments using reference ICs formed by human IgG1 and IgG3 mAbs, the C3b/iC3b deposition onto was completely abolished when complement activation was performed in C1q-depleted serum, as well as in C4-depleted serum or normal serum containing EDTA, but less affected in FB-depleted serum (**Figure 5C**). This indicates that the classical pathway is essential to initiate complement activation by human IgG1 and IgG3 ICs, while the alternative pathway may further amplify (but is not essential for) this activation.

### The Alternative Pathway Is Sufficient for Complement Activation of THSD7A ICs

**Figure 6:**
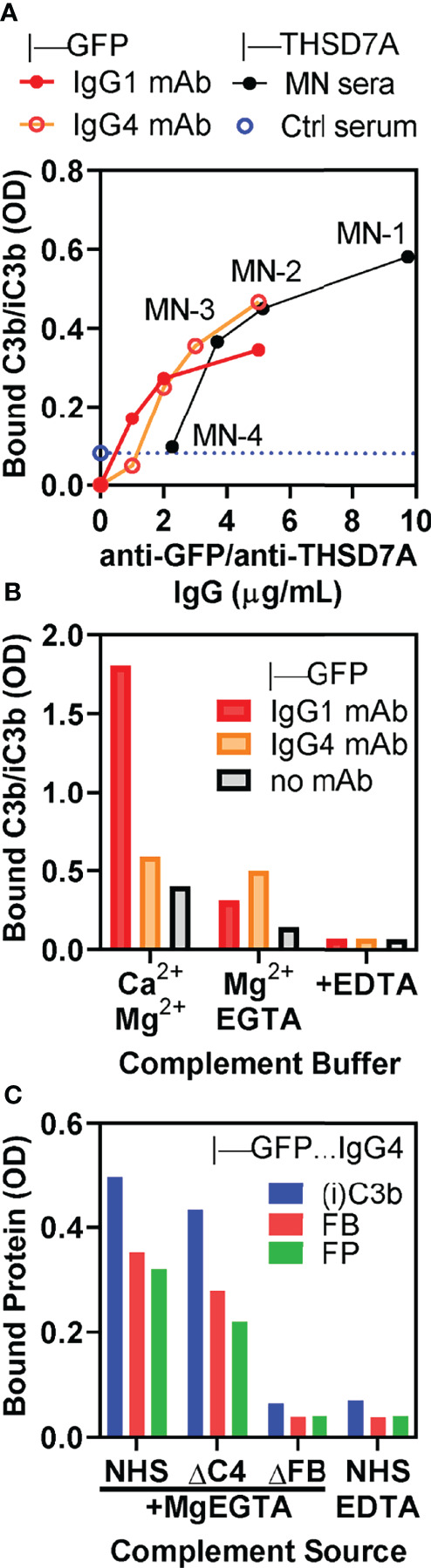
*In vitro* complement activation by THSD7A ICs in MgEGTA buffer, which inhibits the classical and lectin pathways. **(A)** Concentration dependence of C3b/iC3b deposition on ICs formed by anti-THSD7A antibodies (black circles) from MN-1, MN-2, MN-3 and MN-4 sera (all diluted 1/25) bound to immobilized THSD7A or by anti-GFP human IgG1 (red circles) and anti-GFP IgG4 (orange circles) mAbs bound to immobilized GFP. **(B)** C3b/iC3b deposition on ICs formed by IgG1 mAb (1 μg/mL) and IgG4 mAbs (3 μg/mL) bound to immobilized GFP. Complement activation was performed using 10% NHS diluted in buffer containing Ca^2+^ and Mg^2+^, MgEGTA, or EDTA. Inhibition of classical and lectin pathways by MgEGTA reduced C3b/iC3b binding to IgG1 ICs by >80% but had minimal effect on C3b binding to IgG4 ICs. **(C)** The deposition of C3b/iC3b (blue), factor B (FP, red) and properdin (FP, green) on ICs formed by anti-GFP IgG4 mAb (3 μg/mL) was completely abolished when using FB-depleted serum or normal serum containing EDTA as complement source, but largely unchanged in C4-depleted serum.

We further corroborated the role of the alternative pathway by performing complement activation in normal human serum containing MgEGTA, which inhibits the classical/lectin pathways while the alternative pathway remains active. Under these conditions, we observed concentration-dependent C3b/iC3b binding in direct proportion with the amount of anti-THSD7A IgG in ICs formed by MN1-MN3 sera (**Figure 6A**). Reference ICs formed by IgG1 and IgG4 anti-GFP mAbs bound to GFP exhibited a very similar concentration dependent C3b/iC3b binding in MgEGTA, suggesting that the major determinant of alternative pathway activation is the density of IgG4 in ICs rather than the IgG4 specificity for antigen. In control experiments, we further established that C3b/iC3b deposition onto IgG4-GFP ICs was not affected by inhibition of the classical/lectin pathways, while C3b/iC3b binding to IgG1-GFP ICs was inhibited by more than 80% (**Figure 6B**). Furthermore, when complement activation was performed in the presence of MgEGTA, the binding of C3b/iC3b, factor B and properdin to IgG4-GFP ICs was completely abolished in FB-deficient serum, but not affected in C4-deficient serum (**Figure 6C**). This result shows that a functional alternative pathway is necessary and sufficient for complement activation by IgG4.

### Proteomic Identification of Complement Proteins in Microdissected Glomeruli

We also performed a preliminary LC-MS analysis of complement proteins in microdissected glomeruli from patients with THSD7A-associated MN, as compared to those from PLA2R1-associated MN (**Supplemental Figure 1A**). Whereas the average iBAQ intensity values were essentially the same for heavy chains of all four IgG subclasses, the average intensities for C3, C4A and C4B, and terminal pathway components (C5, C6, C7, C8, and C9) were consistently about 2-fold lower in THSD7A-MN (**Supplemental Figure 1B**), implying less complement activation occurs in THSD7A-associated MN compared to PLA2R1-associated MN. The alternative pathway component factor B and a specific inhibitor of this pathway, factor H, were detected with similar intensities in both forms of MN. Lectin pathway components e.g. MASP1, were detected sporadically and with low intensity. Curiously, classical pathway components— such as C1q subunits, C1r and C1s— were detected in almost all cases in both forms of MN. Proteomic identification of C1q, in apparent contradiction with the negative C1q staining in MN biopsies as well as with our results (**Figure 4F**). This suggests that C1q may in fact be bound to ICs but becomes “concealed” (undetectable by anti-C1q antibodies) due to C3b/iC3b deposition on ICs, as previously reported (34).

## Discussion

Whereas podocyte injury by C5b-9 is considered a major effector mechanism of ICs causing proteinuria in MN, there is less agreement about which subclasses of autoAbs and which complement activation pathways are involved. In this study, we have measured quantitatively the serum levels of anti-THSD7A subclasses and assessed their ability to fix complement *in vitro*.

For quantitative analyses of IgG subclasses, we generated calibration curves using recombinant human mAbs bound to their target antigen. Since this ensures oriented binding of the IgG mAbs, similar to binding of anti-THSD7A IgG to coated THSD7A, we propose that this assay format provides more accurate results than using human IgG1-4 myeloma proteins directly absorbed onto plastic wells, an approach previously used to quantify anti-PLA2R IgG subclasses (35). We found that IgG4 was the dominant class of anti-THSD7A autoAbs in the majority (14/16, 88%) of sera analyzed in this work. IgG4 antibodies feature several unusual properties (36). They undergo Fab arm exchange, which accounts for their functional monovalency and inability to form large precipitating ICs (37). With regard to its effector properties, IgG4 is generally considered unable to fix the complement since it cannot bind C1q to initiate the classical pathway (22–24). In addition, by competing with IgG1 and IgG3 for binding to (auto)antigen, IgG4 can inhibit the activation of classical complement pathway by other IgG subclasses (38).

Under the conditions used in our functional complement activation assay (MN sera diluted 1/25), we observed significant deposition of complement activation products only on ICs formed by three sera with the highest levels of THSD7A autoAbs (MN1-MN3), which were all dominant IgG4. Compared to reference ICs formed by IgG1 and IgG3 mAbs, THSD7A ICs activated complement to a lesser extent and only at relatively high concentrations of IgG autoAbs (at least 3.7 μg/mL). The detection of C3b, factor B and properdin bound to THSD7A ICs formed by MN1-MN3 sera is consistent the formation of a stabilized C3 convertase of the alternative pathway, C3bBbP. Bound C4 was also detected in the absence of C1q, which suggests that either the lectin pathway is activated, or that C3b/iC3b deposition conceals bound C1q (34). Finally, bound C5b-9 was detected in significant amounts only on THSD7A ICs formed by MN-1 serum, indicating that the downstream activation of the terminal pathway requires even higher concentrations of anti-THSD7A IgG (5-10 μg/ml) than complement activation at the level of C3.

For all complement-fixing MN sera, the C3b/iC3b binding to THSD7A-IgG4 complexes was completely abolished in FB-depleted serum but was largely unchanged in C1q-depleted sera, indicating that the alternative pathway is essential for complement activation, while the classical pathway is dispensable. For two sera (MN2 and MN3), C3b/iC3b deposition was partially inhibited (by about 35%-50%) in C4-depleted serum, which provides circumstantial evidence of lectin pathway activation. Nonetheless, the same sera supported C3b/iC3b fixation in the presence of MgEGTA, implying that the classical and lectin pathways are dispensable. Taken together, these results show that (a) the alternative pathway is necessary and sufficient for complement activation by THSD7A ICs, and (b) the lectin pathway may contribute to but is not critical for complement activation by anti-THSD7A IgG4. Prior studies using model recombinant human mAbs have shown that ICs formed at high IgG4 concentration and high epitope density can activate complement *via* the alternative pathway (25–27). Our results are the first to show that these findings further extend to naturally occurring human IgG4 autoAbs from patients.

Complement activation products are detected on biopsy in most cases of THSD7A-associated MN (39), although the intensity of staining is often weaker than what is typically seen in PLA2R-associated MN. While this is attributable to ICs formed to anti-THSD7A autoAbs, we cannot rule out that “second-wave” antibodies such as those to cytoplasmic neoantigens upregulated in disease also contribute complement activation (40). In addition, glomerular complement activation in MN may also occur independently of ICs because of local complement dysregulation. A putative mechanism is the impaired recruitment of factor H, a major regulator of the alternative pathway, secondary to the loss of heparan sulfate from the GBM in MN (41). Also, the dysregulation of the alternative pathway may be the result of autoAbs to factor H, which have been reported to occur in as many as 3% of patients with PLA2R-associated MN (42).

Given the relatively weak ability of anti-THSD7A autoAbs to activate complement (especially the terminal pathway) found in our study, we speculate that there may be additional mechanisms by which injury occurs in this form of MN. Anti-THSD7A may have direct function-blocking effects that directly lead to breakdown of the glomerular filtration barrier and consequent proteinuria, since THSD7A localizes directly basal to the slit diaphragm protein nephrin and may thereby stabilize podocyte adhesion and structure (43). Supporting this view, mice passively immunized with human anti-THSD7A autoAbs, as well as mice injected with rabbit anti-THSD7A IgG, developed proteinuria associated with subepithelial ICs and histologic features of MN in the absence of significant glomerular C3b/iC3b deposition (44, 45).

An unexpected finding was the detection of IgA1 anti-THSD7A in 4/16 (25%) of MN sera. To our knowledge, this is the first instance of a podocyte antigen implicated in MN being targeted by IgA autoAbs. Positive glomerular IgA staining has been reported in about 40% of biopsies from THSD7A-associated MN, compared to ~10% of cases of PLA2R-associated MN (15, 39). In our study cohort, positive (1+) glomerular IgA staining on the renal biopsy was found in only one patient, who had the second highest serum titer of circulating IgA anti-THSD7A. This suggest that serum IgA anti-THSD7A autoAbs may deposit in glomeruli, at least in some cases of MN.

Our study has several limitations. With regard to complement activation, the ICs formed *in vitro* represent a simplified 2D model for subepithelial ICs, which may not fully reproduce the complex *in vivo* milieu, comprising the surrounding GBM and the adjacent podocytes. The essential role of the alternative pathway in complement activation by anti-THSD7A IgG4 remains to be corroborated *in vivo* using animal models. As discussed above, mice passively immunized with anti-THSD7A patient sera only exhibit IgG4 but not C3b/iC3b deposition in glomeruli, despite developing proteinuria and histologic features of MN. The lack of complement activation in this mouse model is likely due to insufficient surface density of human IgG4 deposited in mouse glomeruli and/or sub-optimal activation of mouse complement by human antibodies. Nonetheless, we have shown that the alternative pathway is essential for glomerular complement deposition and proteinuria in another mouse model of MN induced by active immunization with α3NC1 (46). This model features subepithelial ICs containing predominantly mouse IgG1, which recapitulates human IgG4 regarding its inability to bind C1q and activate the classical pathway (47).

In summary, our results show that predominantly IgG4 anti-THSD7A autoAbs have the ability to activate complement *via* the alternative pathway, albeit only at high IgG4 surface density. Additional mechanisms yet to be defined may contribute to pathogenic complement activation in THSD7A-associated MN. It is also possible that the pathogenicity of anti-THSD7A autoAbs may be independent of, or only partly mediated by their ability to fix complement. Further studies are required to determine the relative contribution of complement-independent and complement-dependent mechanisms of podocyte injury in THSD7A-associated MN.

## Data Availability Statement

The raw data supporting the conclusions of this article will be made available by the authors, without undue reservation.

## Ethics Statement

The studies involving human participants were reviewed and approved by the respective Institutional Review Boards at each institution. The patients/participants provided their written informed consent to participate in this study.

## Author Contributions

D-BB conceived the idea, designed the research studies, performed data analysis, and wrote the original draft. PM, AS, and TC performed experiments and collected, assembled, analysed, and interpreted data. TC and LB provided critical reagents/samples and curated data. All authors contributed to editing, reviewed, and approved the final manuscript.

## Funding

This work was supported by pilot grants funded by the Meharry Research Centers in Minorities Institutions grant U54MD007586 from the National Institute on Minority Health and Health Disparities of the National Institutes of Health (PM and D-BB). Support was also provided by the award W81XWH-20-1-0698 (grant number LR190150) from the Lupus Research Program of the US Department of Defense (D-BB) and institutional funding for the Glomerular Disease Center, Boston Medical Center (LB).

## Conflict of Interest

The authors declare a potential conflict of interest and state it below. LB reports being a co-inventor on the U.S. patent “Diagnostics for Membranous Nephropathy” and receives royalty income through Boston University. LB has served on advisory boards on the topic of MN and other glomerular diseases for Visterra, Ionis, Alexion, and Novartis, and receives royalties from UpToDate for topics related to MN. TC is employed by Arkana Laboratories, Little Rock, AR.

The remaining authors declare that the research was conducted in the absence of any commercial or financial relationships that could be construed as a potential conflict of interest.

## Publisher’s Note

All claims expressed in this article are solely those of the authors and do not necessarily represent those of their affiliated organizations, or those of the publisher, the editors and the reviewers. Any product that may be evaluated in this article, or claim that may be made by its manufacturer, is not guaranteed or endorsed by the publisher.

## Abbreviations

AP, alternative pathway; FB, factor B; FP, properdin; GFP, green fluorescent protein; GVB, gelatin-veronal buffer; MBL, mannose binding lectin; MN, membranous nephropathy; NHS, normal human serum; PLA2R, phospholipase A2 receptor; TCC, terminal complement complex (C5b-9); THSD7A, thrombospondin type-1 domain-containing 7A.
