# Supplementary material for: The Alternative Pathway Is Necessary and Sufficient for Complement Activation by Anti-THSD7A Autoantibodies, Which Are Predominantly IgG4 in Membranous Nephropathy
Source: Front Immunol. 2022 Jul 7;13:952235. doi: 10.3389/fimmu.2022.952235 (PMC9301376; doi:10.3389/fimmu.2022.952235)
Supplement: Supplementary file 1 [file DataSheet_1.docx]

Supplementary Material

# Supplementary Methods

## Preparation of laser-capture microdissected glomeruli for mass spectrometry (MS)

Kidney biopsies from patients with THSD7A-associated MN (N=3) or PLA2R1-associated MN (N=8) were used to isolate laser-capture microdissected glomeruli for mass spectrometry. The patients characteristics are summarized in Supplementary Table 1, and the results of the immunofluorescence staining on the renal biopsy are shown in Supplementary Table 2. Microdissected glomeruli were lysed in 2% sodium dodecylsulfate and 0.1 M dithiothreitol at 99 °C for 1 hour and processed by filter-assisted sample preparation. The clarified lysate was transferred onto Vivacon 500 concentrators (molecular weight cutoff of 30 kDa; Sartorius, Gottingen, Germany). Sodium dodecylsulfate was removed by repeat washes with 8 M urea in 0.1 M Tris/Cl, pH 8.5, and the samples were alkylated with 0.05 M iodoacetamide. Iodoacetamide was removed by 3 washes with 8 M urea/0.1 M Tris/Cl, pH 8.5, followed by 3 washes with 0.05 M ammonium bicarbonate. Proteins were digested with trypsin (sequencing grade, Promega, Madison WI) at a 40:1 w/w ratio at 37 °C for 16 hours. Peptides were collected by centrifugation and desalted on C18-Stage tips (Thermo-Fisher Scientific, Waltham, MA).

## Mass spectrometry

Digested peptides were analyzed by nanoscale liquid chromatography coupled to tandem MS using a Thermo Orbitrap Fusion Lumos mass spectrometer (Thermo Fisher Scientific). Peptides were loaded onto a reverse phase trap column (IntegraFrit, New Objective, Littleton, MA) containing 2.5 μm Waters XSelect CSH resin (Waters Corporation, Milford, MA) coupled to a 150 mm × 0.075 mm analytical column containing the same reverse phase resin as used in the trap. A nanoACQUITY UPLC system (Waters Corporation, Milford, MA) was then used to generate a 60-minute gradient from 98:2 to 60:40 buffer A/buffer B ratio (buffer A: 0.1% formic acid, 0.5% acetonitrile; buffer B: 0.1% formic acid, 99.9% acetonitrile). Peptides were eluted from the column with an integrated spray tip (PicoFrit, New Objective) and ionized by electrospray (2.0 kV) followed by MS/MS analysis using higher energy collision–induced dissociation. Survey scans of peptide precursors were performed at 240,000 resolution (at 400 m/z) with a 5 × 10^5^ ion count target. Tandem MS was performed by isolation at 1.6 Th with the quadrupole, higher energy collision–induced dissociation fragmentation with a normalized collision energy of 30 eV, and rapid scan MS analysis in the ion trap. The obtained MS/MS data were searched against the most recent UniProt human database containing both the Swiss-Prot and the TrEMBL entries using MaxQuant (Max Planck Institute of Biochemistry). The false discovery rate was set at 1% for the peptide-to-spectrum matches. Normalized intensity-based absolute quantification (iBAQ) values from MaxQuant were used for quantitation. iBAQ distributions for each sample were normalized based on median log_2_ iBAQ value to control for differences in loading.

# Supplementary Figures and Tables

## Supplementary Tables

Supplementary Table 1. Demographic and clinical characteristic of MN patients

Values indicate counts (%) or median (range).

| Patient Characteristics | THSD7A-associated MN | PLA2R1-associated MN |
| --- | --- | --- |
| Number of cases | 3 | 8 |
| Sex: M/F | 2 (67%)/1 (33%) | 3 (37%) /5 (63%) |
| Age (yrs) | 51 (27, 67) | 60 (27, 77) |
| Nephrotic Proteinuria | 3 (100%) | 7 (87%) |
| Serum Albumin (g/dL) | 1.89 (1.8, 2.3) | 2.1 (1.1, 3.3) (N=4) |
| Serum Creatinine |  |  |

Supplementary Table 2. The results of immunofluorescence staining in the renal biopsies analyzed in the proteomics study

| MN Patient # | IgG | IgA | IgM | C3 | C1q |
| --- | --- | --- | --- | --- | --- |
| THSD7A-1 | 2+ | 0 | 0 | 1+ | 0 |
| THSD7A-2 | 2+ | 1+ | 0 | Trace | 0 |
| THSD7A-3 | 3+ | 0 | 0 | 0 | 0 |
| PLA2R-1 | 2+ | 0 | 1+ | 0 | 0 |
| PLA2R-2 | 3+ | 0 | 0 | 2+ | 0 |
| PLA2R-3 | 3+ | 0 | 0 | 2+ | 0 |
| PLA2R-4 | 3+ | 0 | 0 | 2+ | 0 |
| PLA2R-5 | 3+ | 0 | 0 | 3+ | 0 |
| PLA2R-6 | 3+ | Trace | 0 | 2+ | 0 |
| PLA2R-7 | 3+ | 2+ | 0 | 1+ | 0 |
| PLA2R-8 | 3+ | 0 | 0 | 1+ | 0 |

## Supplementary Figures


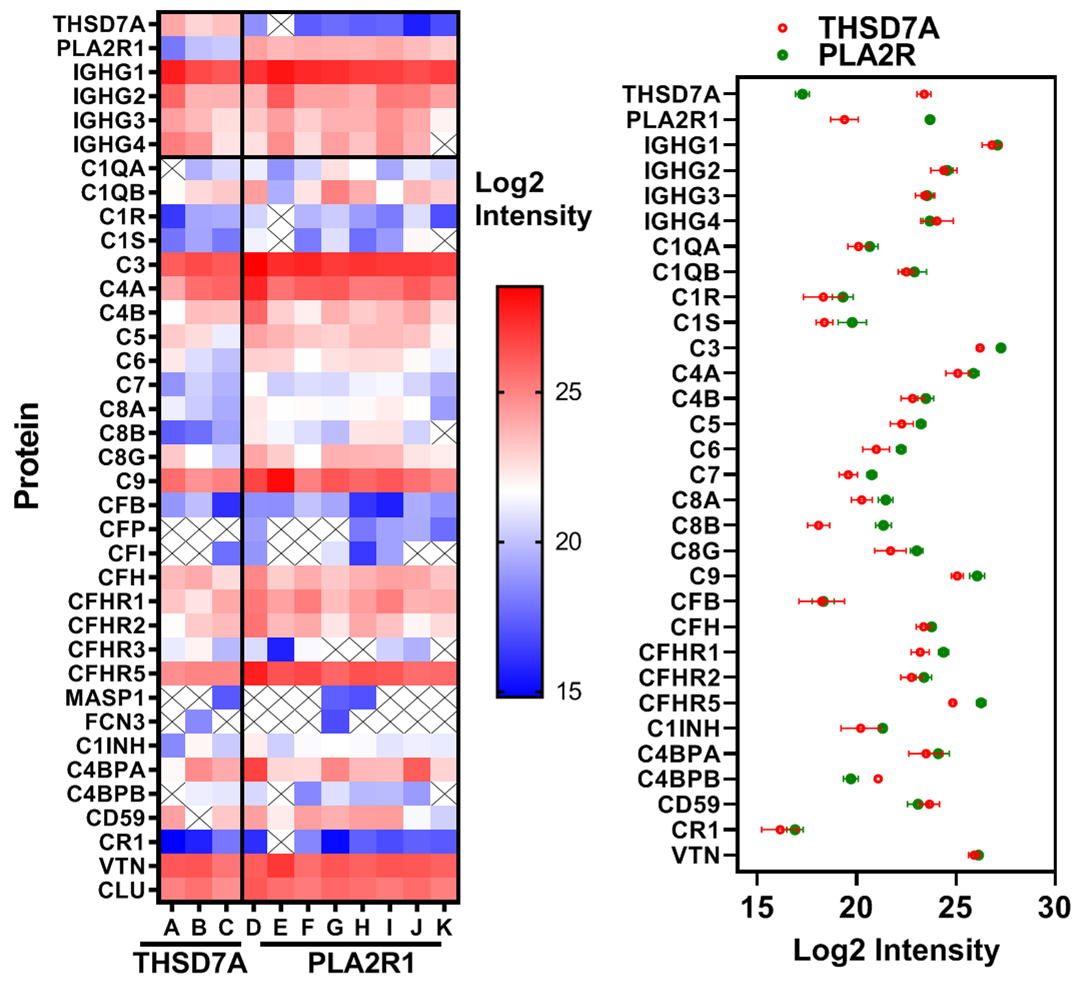


**Figure S1.** **Proteomic identification of complement proteins in THSD7A-associated MN**.
A. Heat map showing log2 iBAQ intensity values for complement proteins from laser captured micro-dissected glomeruli samples analyzed by LC-MS. A color scale for the log2 intensity values is shown on the right. Missing values are indicated by an X symbol. B. Plot depicting the mean ± SEM of log2 iBAQ intensity values for complement proteins identified in THSD7A-associated MN (red) and PLA2R1-associated MN (green).
